# Comparison Between the Protective Effect of Isoflurane and Propofol on Myocardium During Coronary Artery Bypass Grafting: A Systematic Review and Meta-Analysis of Randomized Controlled Trials

**DOI:** 10.21470/1678-9741-2021-0424

**Published:** 2024-04-15

**Authors:** Qi Bao, Min Lei, Dongju Xiao, Junran Xie

**Affiliations:** 1 Department of Anesthesiology, Sir Run Run Shaw Hospital, School of Medicine, Zhejiang University, Zhejiang, People’s Republic of China

**Keywords:** Cardiac Surgery, Isoflurane, Meta-Analysis, Propofol, Troponin I

## Abstract

**Objective:**

Intravenous non-volatile anaesthetics like propofol are commonly used in cardiac surgeries across several countries. Volatile anaesthetics like isoflurane may help in protecting the myocardium and minimize ischaemia-reperfusion injury. Hence, we did this review to compare the cardioprotective effect of isoflurane and propofol among patients undergoing coronary artery bypass grafting (CABG).

**Methods:**

We conducted a search in the databases Medical Literature Analysis and Retrieval System Online (or MEDLINE), Embase, PubMed Central®, ScienceDirect, Google Scholar, and Cochrane Library from inception until April 2021. We carried out a meta-analysis with random-effects model and reported pooled risk ratio (RR) or standardized mean difference (SMD) with 95% confidence interval (CI) depending on the type of outcome.

**Results:**

We analysed 13 studies including 808 participants. Almost all were low-quality studies. For cardiac index, the pooled SMD was 0.14 (95% CI: -0.22 to 0.50); for cardiac troponin I, pooled SMD was 0.10 (95% CI: -0.28 to 0.48). For mortality, the RR was 3.00 (95% CI: 0.32 to 28.43); for MI, pooled RR was 1.58 (95% CI: 0.59 to 4.20); and for inotropic drug use, pooled RR was 1.04 (95% CI: 0.90 to 1.21). For length of intensive care unit stay, the pooled SMD was 0.13 (95% CI: -0.29 to 0.55), while pooled SMD for mechanical ventilation time was -0.02 (95% CI: -0.54 to 0.51).

**Conclusion:**

Isoflurane did not have significant cardioprotective effect compared to propofol following CABG. Hence, the anaesthetists need to check some viable alternatives to manage these patients and reduce the rate of postoperative complications.

## INTRODUCTION

**Table:** 

Abbreviations, Acronyms & Symbols	
CABG	= Coronary artery bypass grafting
CI	= Confidence interval
EIC	= Elective isolated coronary artery bypass grafting
I	= Isoflurane
ICU	= Intensive care unit
MI	= Myocardial infarction
NR	= Not reported
P	= Propofol
RCTs	= Randomized controlled trials
RoB 2	= Cochrane risk-of-bias tool for randomized controlled trials
RR	= Risk ratio
SD	= Standard deviation
SE	= Standard error
SMD	= Standardized mean difference

Management of coronary artery disease patients has undergone several recent advances, especially the surgical treatment, due to newer and innovative techniques and anaesthetic protocols in the coronary artery bypass grafting (CABG). Despite these advances, myocardial damage during the surgery still remains as an inevitable threat^[1-3]^. Myocardial ischaemia-reperfusion injury is one complication that commonly occurs during and after CABG. It leads to serious and marked myocardial dysfunction, probably causing myocardial infarction (MI) and hospitalization for a prolonged period of time^[4]^.

Pharmacological management has become an attractive concept over the years to protect the myocardium and prevent these types of serious injuries. Volatile anaesthetics like isoflurane may help in protecting the myocardium and minimize the ischaemia-reperfusion injury or might have a preconditioning (treatment before an ischaemic event) effect on the myocardium. Such cardioprotective effects have been demonstrated in both human and animal models^[5-7]^. The mechanism of action of isoflurane for myocardial protection and preconditioning has been studied extensively over the years. The possible theories proposed were the opening of mitochondrial potassium adenosine triphosphate channels^[8]^, increase in mitochondrial reactive oxygen species^[9]^, and translocation or activation of the tyrosine kinases, protein kinase C, and p38 mitogen-activated protein kinase^[10]^. These supposedly act by decreasing the mitochondrial and cytosolic calcium loadings. Isoflurane can also suppress the neutrophils activation and neutrophil-endothelium interaction that is responsible for the myocardial dysfunction^[11]^.

Intravenous non-volatile anaesthetics like propofol are commonly used in cardiac surgeries across several countries. However, several trials have explored the use of isoflurane and compared various endpoints or surrogate markers with the propofol for their role on myocardial protection during cardiac surgeries^[12-15]^. Nonetheless, most of these studies have been underpowered to determine a significant cardioprotective effect with respect to all the outcomes. Hence, we did this systematic review and meta-analysis of randomized controlled trials (RCTs) comparing the cardioprotective role of isoflurane with propofol during CABG.

## METHODS

### Eligibility Criteria

#### Study Design

We have included only RCTs (parallel arm individual randomized or cluster RCTs) for the review. Full-text articles or abstracts were included while the unpublished literature was excluded.

#### Participants

We have included the studies conducted in patients undergoing CABG.

#### Intervention

Studies that directly compared the effectiveness of isoflurane against propofol as the anaesthetic to perform CABG were included.

#### Outcome Measures

- Mortality
- MI
- Postoperative cardiac index
- Cardiac troponin I
- Inotropic drug use
- Mechanical ventilation time
- Length of intensive care unit (ICU) stay

We have included the studies reporting any of the abovementioned outcomes in both the arms.

### Search Strategy

A comprehensive, systematic, and extensive search was conducted in electronic databases such as Medical Literature Analysis and Retrieval System Online (or MEDLINE), Embase, PubMed Central®, ScienceDirect, Google Scholar, and Cochrane Library. We selected the terms required for the search during the protocol stage itself. We used both the medical subject headings (or MeSH) and free-text words while performing the search in these databases. The terms used in our search strategy were as follows: “Isoflurane”, “Propofol”, “Volatile Anaesthetics”, “Non-volatile anaesthetics”, “Cardiac Surgery”, “Myocardial Infarction”, “Randomized Controlled Trials”, “Coronary Artery Bypass Grafting”, “Coronary Artery Disease”, and “Cardioprotective Effect”. The set of keywords and their synonyms were used for search using appropriate truncations, wildcards, and proximity searching. Search also was conducted for key concepts using corresponding subject headings in each database. The final search was carried out by combining the individual search results using appropriate Boolean operators (“OR” and “AND”). The search was narrowed down using the available filters on type of studies. We restricted the search from inception of the databases to April 2021 and published in English language only. Bibliographies of the retrieved articles are also hand-searched to identify any articles missed during the database search.

### Study Selection Process

This process has involved three stages:

**Step 1:** Two independent investigators have performed primary screening of title, abstract, and keywords by executing the literature search. Full-text articles were retrieved for the studies shortlisted based on the eligibility criteria.

**Step 2:** Full-text articles of these retrieved studies were screened by the same two investigators and assessed against the eligibility criteria of the review. Studies that satisfied all the eligibility criteria with respect to design, participants, exposure, and outcome were included.

**Fig. 1:**
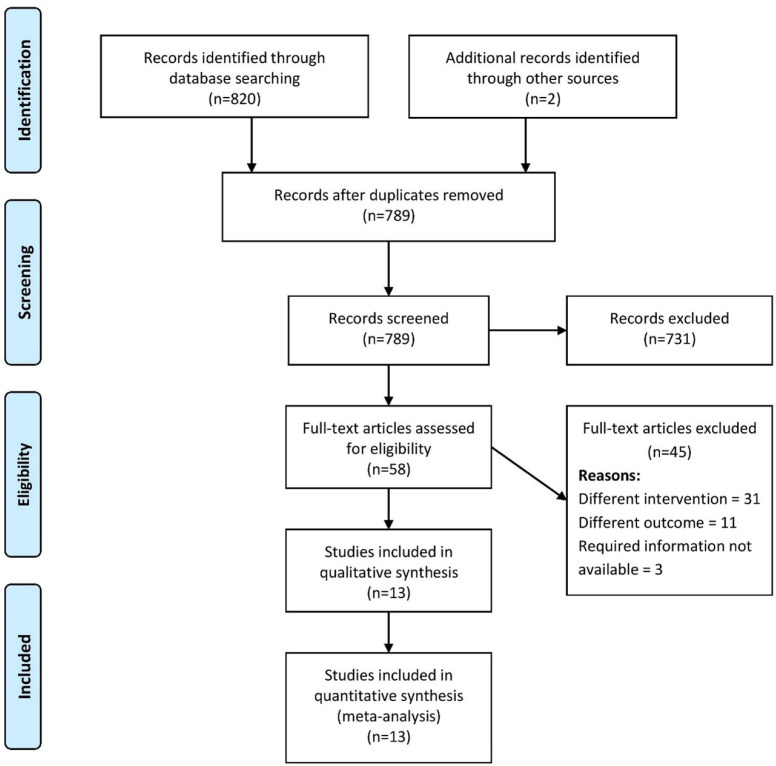
Preferred Reporting Items for Systematic Reviews and Meta-Analyses (or PRISMA) flowchart.

**Step 3:** Disagreements during the screening process between the investigators were resolved and final consensus on inclusion of studies was reached with the help of another investigator. Preferred Reporting Items for Systematic Reviews and Meta-Analyses (or PRISMA) flowchart was used to clearly represent the screening and selection process (Figure 1).

### Data Collection Process and Management

Data was extracted manually from the included studies using a structured data extraction form, developed and pilot tested during the protocol stage itself. Data extracted using the form were as follows: general information about the article such as author and year of publication; information related to methods section such as study design, setting, sample size, randomization details, study participants, inclusion & exclusion criteria, outcome assessment method, and quality-related information; and information related to outcome. Data was entered by the investigator and the entry was double-checked by secondary investigators for correct entry.

### Risk of Bias Assessment

Primary and secondary authors were assigned the responsibility to evaluate the risk of bias amongst the final included studies. Cochrane risk-of-bias tool for randomized controlled trials (RoB 2)^[16]^ was used to assess the bias risk under the following domains:

**Domain 1:** Bias risk arising from the process of randomization.

**Domain 2:** Bias risk due to deviation from the intended intervention.

**Domain 3:** Bias risk arising due to missing data on outcomes.

**Domain 4:** Bias risk in the measurement of outcome.

**Domain 5:** Bias risk in the selection of reported result.

Based on the rating obtained from these domains, each study was classified as having “low bias risk”, “high bias risk”, and “some concerns” on the quality of evidence.

### Statistical Analysis

Meta-analysis was executed using the software STATA version 14.2 (StataCorp, CollegeStation, Texas, United States of America). For the dichotomous outcomes such as mortality, MI, and inotropic drug use, number of events and participants in each group were entered to obtain the pooled effect estimate in terms of odds ratio and visually represented through forest plot. For continuous outcomes such as cardiac index/output, cardiac troponin I, mechanical ventilation time, and length of hospital stay, mean, standard deviation, and total sample size were obtained for both groups. The pooled effect was interpreted in terms of mean difference or standardized mean difference (SMD) with 95% confidence interval (CI). We used the random effects model with inverse variance method to calculate the weightage of individual studies^[17]^. Evidence of between-study variance due to heterogeneity was assessed through chi-squared test of heterogeneity and I2 statistics to quantify the inconsistency. We also performed sensitivity analysis to assess the robustness of results by removing the studies one by one and checking for any significant variation in the results. Publication bias was assessed using funnel plot and statistically inferred using Egger’s test.

## RESULTS

### Study Selection Process

We found 820 records through the systematic literature search and deemed 58 of those studies relevant for full-text retrieval. We also retrieved full-texts for two articles obtained through manual searching of the bibliographies in the retrieved studies. During the second screening stage, 13 studies with 808 participants met the eligibility criteria and were included in the analysis (Figure 1)^[12-15,18-26]^.

### Characteristics of Studies Included

Characteristics of the studies are described in Table 1. All the studies were RCTs. Most of the studies were conducted in European countries such as Ireland, Germany, and United Kingdom, followed by Asian countries such as China and India. In total, 808 participants were found in the included studies with sample size ranging from 20 to 236. The mean age of the study participants has ranged from 53 to 68 years. All the studies had participants undergoing elective isolated CABG. In total, 11 studies had reported on mechanical ventilation time, eight studies each have reported on cardiac index and MI, seven studies have reported on mortality, cardiac troponin, and length of ICU stay, and six studies have reported on inotropic drug use.

**Table 1:** Characteristics of the included studies (N=13).

Study nº	First author and year	Country	Study design	Sample size (I *vs.* P)	Type of surgery	Aortic cross-clamping	Mean age (years)	Outcomes assessed
1	El-Shobaki et al., 2002	Egypt	RCT	I=25	EIC	NR	NR	Cardiac index, length of ICU stay, mechanical ventilation time
				P=25				
2	Engoren et al., 1998	United States of America	RCT	I=35	EIC	NR	61	In-hospital mortality, MI, length of ICU stay, mechanical ventilation time
				P=35				
3	Flier et al., 2010	Netherlands	RCT	I=41	EIC	53	67	Cardiac index, cardiac troponin, in-hospital mortality, MI, inotropic drug use, length of ICU stay, mechanical ventilation time
				P=43				
4	Huang et al., 2011	Italy	RCT	I=30	EIC	NR	61	Cardiac index, cardiac troponin, in-hospital mortality, MI, inotropic drug use, length of ICU stay, mechanical ventilation time
				P=30				
5	Imantalab et al., 2012	Iran	RCT	I=20	EIC	41	NR	Cardiac troponin, mechanical ventilation time
				P=20				
6	Kendall et al., 2004	United Kingdom	RCT	I=10	EIC	NR	I=58.1	Cardiac troponin, MI, inotropic drug use, mechanical ventilation time
				P=10			P=68.1	
7	Kottenberg et al., 2011	Germany	RCT	I=19	EIC	72	65	Cardiac troponin, in-hospital mortality
				P=19				
8	Parker et al., 2004	Australia	RCT	I=118	EIC	NR	66	Cardiac index, in-hospital mortality, MI, inotropic drug use, length of ICU stay, mechanical ventilation time
				P=118				
9	Phillips et al., 1994	Ireland	RCT	I=31	EIC	NR	60	Cardiac index
				P=33				
10	Sorbara et al., 1995	Italy	RCT	I=15	EIC	67	60	Cardiac index, mechanical ventilation time
				P=15				
11	Tempe et al., 2011	India	RCT	I=20	EIC	NR	I=53	Cardiac index, in-hospital mortality, MI, inotropic drug use, mechanical ventilation time
				P=20			P=54	
12	Xia et al., 2006	China	RCT	I=18	EIC	84	64	Cardiac index, cardiac troponin, MI, inotropic drug use, length of ICU stay, mechanical ventilation time
				P=18				
13	Yildrim et al., 2009	Turkey	RCT	I=20	EIC	2	68	Cardiac index, cardiac troponin, in-hospital mortality, MI, length of ICU stay, mechanical ventilation time
				P=20				

EIC=elective isolated coronary artery bypass grafting; I=isoflurane; ICU=intensive care unit; MI=myocardial infarction; NR=not reported; P=propofol; RCT=randomized controlled trial

### Risk of Bias Assessment

Table 2 shows the risk of bias across various domains as per the RoB 2 tool results. We found that all the studies had low risk or some concerns over the randomization process. With respect to the other domains, 10 studies had high risk or some concerns over the deviation of the intended intervention domain, eight studies had high risk or some concerns over the missing outcome data domain, seven studies had high risk of bias over the measurement of outcome domain, and only three studies had high risk of some concerns over the selection of reporting results. Overall, 11 out of 13 studies had high risk of bias, and the other two studies had some concerns.

**Table 2:** Risk of bias assessment (N=13).

Study nº	Author and year	Randomization process	Deviation from intended intervention	Missing outcome data	Measurement of the outcome	Selection of the reported results	Overall
1	El-Shobaki et al., 2002	Low	High	Low	High	High	High
2	Engoren et al., 1998	Low	Some concerns	High	High	High	High
3	Flier et al., 2010	Low	Some concerns	Low	Low	Low	Some concerns
4	Huang et al., 2011	Some concerns	High	Low	Low	Low	High
5	Imantalab et al., 2012	Some concerns	Some concerns	High	Low	Low	High
6	Kendall et al., 2004	Low	High	High	Low	Low	High
7	Kottenberg et al., 2011	Low	High	Some concerns	High	Low	High
8	Parker et al., 2004	Low	Low	High	High	High	High
9	Phillips et al., 1994	Low	Some concerns	High	High	Low	High
10	Sorbara et al., 1995	Low	High	Low	Low	Low	High
11	Tempe et al., 2011	Some concerns	Low	Low	Low	Low	Some concerns
12	Xia et al., 2006	Low	Low	High	High	Low	High
13	Yildrim et al., 2009	Some concerns	High	High	High	Low	High

### Cardioprotective Efficacy of Isoflurane and Propofol Among Patients Undergoing Cardiac Surgery

#### Cardiac Index

**Fig. 2:**
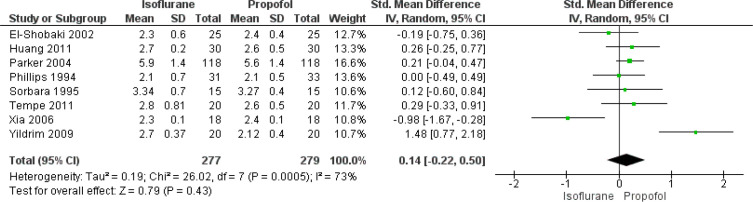
Forest plot showing differences in cardiac index between isoflurane and propofol groups. CI=confidence interval; SD=standard deviation.

In total, eight studies have reported on the effect of isoflurane and propofol on cardiac index of patients undergoing CABG. The pooled SMD was found to be 0.14 (95% CI: -0.22 to 0.50), and this difference was not statistically significant (*P*=0.43) (Figure 2). There was significant heterogeneity among the included studies reporting this outcome (I^2^=73%, *P*<0.001).

#### Cardiac Troponin I

**Fig. 3:**
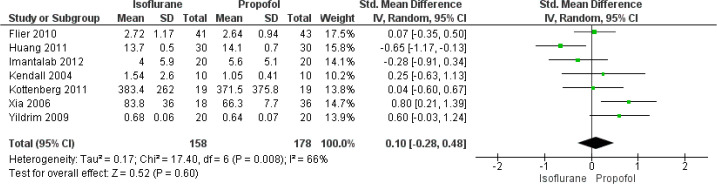
Forest plot showing differences in cardiac troponin between isoflurane and propofol groups. CI=confidence interval; SD=standard deviation.

In total, seven studies have reported on the effect of isoflurane and propofol on cardiac troponin I of patients undergoing CABG. The pooled SMD was found to be 0.10 (95% CI: -0.28 to 0.48), indicating that there was no significant difference in cardiac troponin I between patients receiving isoflurane and propofol during CABG (*P*=0.60) (Figure 3). There was significant heterogeneity among the included studies reporting this functional outcome (I^2^=66%, *P*=0.008).

#### Mortality

**Fig. 4:**
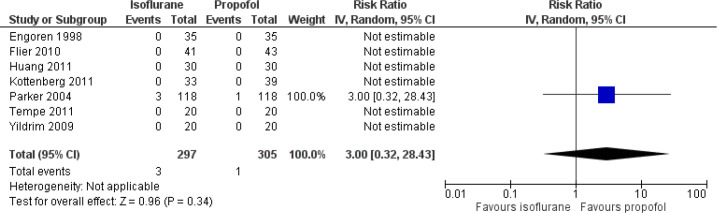
Forest plot showing differences in mortality between isoflurane and propofol groups. CI=confidence interval.

Though seven studies have reported on the mortality rate, only one study had deaths in both isoflurane and propofol groups. The risk ratio (RR) was 3.00 (95% CI: 0.32 to 28.43) (Figure 4). Assessment of heterogeneity was not applicable as only one study had reported deaths in both groups and the rest of the studies showed zero death in both groups.

#### MI

**Fig. 5:**
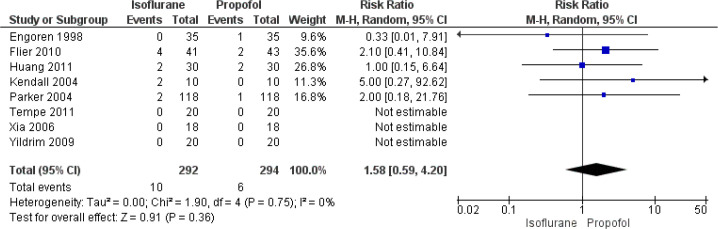
Forest plot showing differences in myocardial infarction between isoflurane and propofol groups. CI=confidence interval.

In total, eight studies have reported on the effect of isoflurane and propofol on the rate of MI following CABG. The pooled RR was 1.58 (95% CI: 0.59 to 4.20) (Figure 5). This indicates that the patients undergoing CABG under the influence of isoflurane have 1.58 times higher risk of having MI when compared to those undergoing CABG under the influence of propofol exposure. However, this association was not statistically significant (*P*=0.36). We found no heterogeneity between the studies reporting the MI rate (I^2^=0%, *P*=0.75).

#### Inotropic Drug Use

**Fig. 6:**
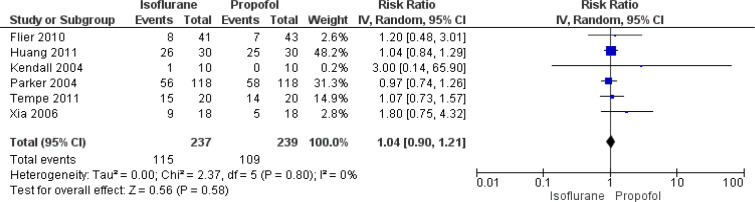
Forest plot showing differences in inotropic drug use between isoflurane and propofol groups. CI=confidence interval.

In total, six studies have reported on the effect of isoflurane and propofol on the rate of inotropic drug use following CABG. The pooled RR was 1.04 (95% CI: 0.90 to 1.21), indicating no significant difference in terms of inotropic drug use between the two groups (Figure 6). We found no heterogeneity between the studies reporting this outcome (I^2^=0%, *P*=0.80).

#### Length of ICU Stay

**Fig. 7:**
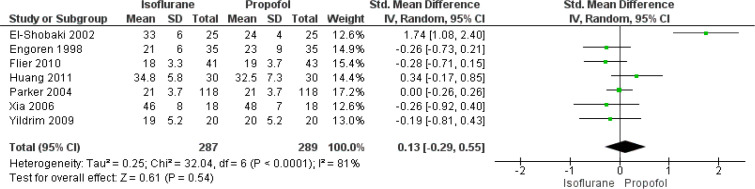
Forest plot showing differences in length of intensive care unit stay between isoflurane and propofol groups. CI=confidence interval; SD=standard deviation.

In total, seven studies have reported on the effect of isoflurane and propofol on length of ICU stay of patients undergoing CABG. The pooled SMD was found to be 0.13 (95% CI: -0.29 to 0.55), indicating that there was no significant difference in length of ICU stay between patients receiving isoflurane and propofol during CABG (*P*=0.54) (Figure 7). There was substantial heterogeneity among the included studies reporting this functional outcome (I^2^=81%, *P*<0.001).

#### Mechanical Ventilation Time

**Fig. 8:**
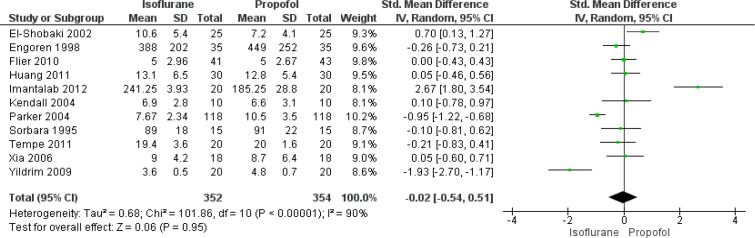
Forest plot showing differences in mechanical ventilation time between isoflurane and propofol groups. CI=confidence interval; SD=standard deviation.

In total, 11 studies have reported on the effect of isoflurane and propofol on time under mechanical ventilation of patients undergoing CABG. The pooled SMD was found to be -0.02 (95% CI: -0.54 to 0.51), indicating that there was no significant difference in mechanical ventilation time between patients receiving isoflurane and propofol during CABG (*P*=0.95) (Figure 8). There was significant heterogeneity among the included studies reporting this functional outcome (I^2^=90%, *P*<0.001).

#### Additional Analysis

**Fig. 9:**
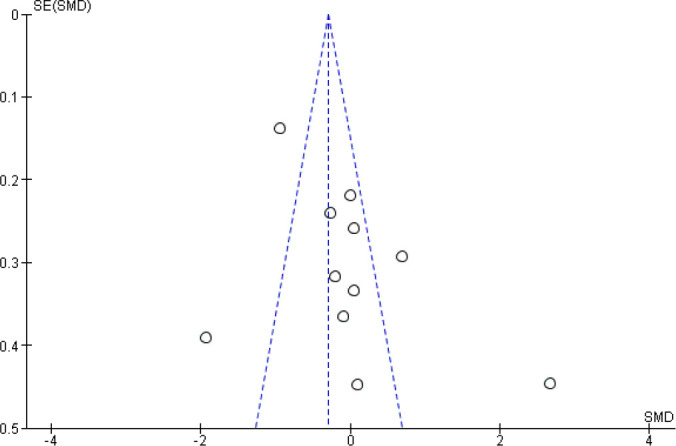
Funnel plot for assessing publication bias. SE=standard error; SMD=standardized mean difference.

Since only the outcome on mechanical ventilation time had enough number of studies to assess the publication bias (> 10 studies), we have visually inspected the funnel plot only for this outcome and found it to be asymmetrical (Figure 9). It was further confirmed by significant Egger’s test (*P*=0.03). Sensitivity analysis has showed that there was no significant variation in the magnitude or direction of any of the outcomes, indicating lack of influence of a single study on the overall pooled estimate.

## DISCUSSION

We did this review to update regarding the cardioprotective efficacy and safety of isoflurane compared to propofol among patients undergoing CABG. We have found 13 studies matching the eligibility of our review, conducted mostly in European and Asian countries. All the studies were RCTs and of lower quality as per RoB 2 tool.

This meta-analysis has provided several results having important clinical implications. In the patients undergoing CABG, isoflurane and propofol had similar mortality rate. This result was in line with the previous meta-analysis conducted in the heterogeneous anaesthetic and surgical settings^[27-30]^. The reason for no difference in mortality could be the poor statistical power of this outcome, as the short-term or in-hospital mortality is a rare event. In fact, the RR, when calculable, were based on small number of events and higher variability. Such results indicate the need for a greater number of trials to clearly dissect the impact of isoflurane and propofol on mortality rates.

We also found that the isoflurane was ineffective in reducing the postoperative cardiac index, cardiac troponin release, MI incidence, need for inotropic drugs, length of ICU stay, and mechanical ventilation time. The findings were in line with previous reviews^[29,30]^, which also found isoflurane to be ineffective compared to propofol in reducing any of the cardiac and postoperative outcomes. However, other volatile anaesthetics like desflurane and sevoflurane were found to cause lesser cardiac depression and preserve the cardiac function compared to propofol, indicating the fact that these volatile anaesthetics (except isoflurane) act as a better alternative with better cardioprotective effect^[30]^. However, the type of surgery, study era, and the aortic cross-clamping time have been found to influence the effect of volatile anaesthetics on major cardiac surrogate endpoints such as cardiac index and cardiac troponin release^[30]^.

Previous reviews have shown that a cardioprotective effect was favoured by the volatile anaesthetics in patients undergoing isolated CABG and shorter aortic cross-clamping times^[29,30]^. However, due to the multiple connections between these variables, multiple subgroup analysis cannot be performed owing to the limitation in the number of studies in each subgroup and cannot find the role in influencing the isoflurane effect on these endpoints. This further reiterates the importance of having a greater number of trials to comprehensively look at the various aspects of cardioprotective effect of isoflurane compared to propofol.

Although the results in our meta-analysis and the previous meta-analytic researches have never showed any beneficial effect of propofol compared to isoflurane or any volatile anaesthetics, some cardioprotective mechanisms have been found in the isolated organs and cells with propofol^[31-34]^. Probably, the cardioprotective effect of propofol might be overwhelmed *in vivo* by the volatile anaesthetic effect.

Our review has certain strengths. The major strength is the rigorous literature search and methodology followed to provide reliable estimates. We included only RCTs conducted in patients undergoing CABG, making the evidence generated for all the outcomes more reliable compared to previous meta-analysis (which also included observational studies). We also performed a comprehensive search of evidence and included studies up to 2021 to make us reach the best possible evidence on the current level of cardioprotective efficacy of both the anaesthetic groups on this topic.

### Limitations

Despite these strengths, our meta-analysis has some limitations. Our results should be interpreted with caution and inferred accordingly, considering the difference in methods and quality across the included studies. In our analysis, we found significant between-study variability (significant chi-squared test for heterogeneity and I2 statistics) for all the continuous outcomes such as cardiac index, cardiac troponin release, mechanical ventilation time, and length of hospital stay. Reason for such high heterogeneity can be attributed to the methodological differences between the included studies such as study design, setting, sample size, type of surgery, and cross-clamping time, and difference in definitions of the outcomes like MI. However, we could not explore these reasons given the limitation in the number of studies to perform additional subgroup analysis or meta-regression. In addition, we found significant publication bias in our review with respect to outcome on mechanical ventilation time, which can limit the credibility of the evidence. In addition, we could not assess the publication bias for the other outcomes due to limitation in the number of studies. We also found that some trials had used total intravenous anaesthetics for induction and for shorter period of anaesthesia maintenance in the isoflurane arm, which might have attenuated the effect obtained in our review^[35-37]^.

Future research should focus on conducting a large scale RCT among the high-risk patients having homogeneous surgical and anaesthesiologic protocols, needed to evaluate the impact of isoflurane alone with the propofol alone. Future RCTs should also strive towards disclosing conclusively the short-term and long-term cardioprotective effects of these drugs following CABG.

## CONCLUSION

To conclude, isoflurane did not have significant cardioprotective effect compared to propofol following CABG. Hence, the anaesthetists need to check some viable alternatives to manage these patients and reduce the rate of postoperative complications.
